# A Scalable Approach to IoT Interoperability: The Share Pattern

**DOI:** 10.3390/s25154701

**Published:** 2025-07-30

**Authors:** Riccardo Petracci, Rosario Culmone

**Affiliations:** School of Science and Technology, University of Camerino, 62032 Camerino, Italy; rosario.culmone@unicam.it

**Keywords:** service composition, design pattern, embedded systems, design-by-contract

## Abstract

The Internet of Things (IoT) is transforming how devices communicate, with more than 30 billion connected units today and projections exceeding 40 billion by 2025. Despite this growth, the integration of heterogeneous systems remains a significant challenge, particularly in sensitive domains like healthcare, where proprietary standards and isolated ecosystems hinder interoperability. This paper presents an extended version of the Share design pattern, a lightweight and contract-based mechanism for dynamic service composition, tailored for resource-constrained IoT devices. Share enables decentralized, peer-to-peer integration by exchanging executable code in our examples written in the LUA programming language. This approach avoids reliance on centralized infrastructures and allows services to discover and interact with each other dynamically through pattern-matching and contract validation. To assess its suitability, we developed an emulator that directly implements the system under test in LUA, allowing us to verify both the structural and behavioral constraints of service interactions. Our results demonstrate that Share is scalable and effective, even in constrained environments, and supports formal correctness via design-by-contract principles. This makes it a promising solution for lightweight, interoperable IoT systems that require flexibility, dynamic configuration, and resilience without centralized control.

## 1. Introduction

The Internet of Things (IoT) comprises a vast network of interconnected devices, including various systems such as computer systems, mobile gadgets, sensors, and actuators. Each device is uniquely identified and can exchange data across the network autonomously [1]. IoT devices have grown to an enormous number of about 30 billion units connected to the Internet, and scientists estimate these numbers may grow to a threshold of 40 billion devices by the year 2025. In the variety of devices, we have an aggregation of about 20 per cent of the total, comprised of short-range sensors and more general devices used in home automation [2]. The installation of these devices is carried out without a structured plan and by the average user at the time of purchase, often resulting in heterogeneous devices. The lack of structured deployment leads to fragmentation, posing challenges for seamless communication. This is because the various devices are designed and developed by different manufacturers, and their interaction is entrusted to other devices or services, which complicates the system design. In sensitive domains such as healthcare, communication and interoperability between devices are crucial, and integrating devices so adept at communicating with each other in a common ecosystem is very complex, if not impossible, due to closed or proprietary protocols. IoT services are present in a wide range, from smart home automation to healthcare applications, and this massive presence of different services has rendered standardization efforts futile [3,4]. In this work, we present a pattern that enables dynamic service composition with formal guarantees of correctness, optimized for embedded environments.

In the contemporary context, the integration of IoT services is being approached through a variety of methodologies. Different IoT cloud actors have a preference for solutions that centralize data. Consequently, users are able to define centralized applications to define the related integrated services. The problem that arises is related to scalability, a feature that cannot be fully achieved through a centralized solution; consequently, there is a move towards computation as close as possible to the edge [5,6]. Often, a prior methodological approach is required in which client and server agree on semantics and format for each service before composition takes place. On the opposite side, one should think in terms of dynamic composition, where the composition of IoT services is a widely studied problem [7,8]. We note that the frameworks OSGi, SM4ALL [9], and WSDL [10] offer a viable solution for service composition [11,12]. This poses a significant challenge to the potential for a decentralized and peer-to-peer integration of services, as the limited memory sizes and computing power of embedded devices can impede the efficacy of these systems.

In our previous work, we introduced “Share: A Design Pattern for Dynamic Composition of IoT Services” [13], which proposed a novel integration model. This work intends to present an extension of our research. Continuing the path started in previous research, we developed an execution emulator to test and validate the effectiveness of the pattern. This emulator was implemented using the LUA programming language, chosen for its lightness and adaptability to resource-limited devices. Furthermore, an investigation was conducted into the Object Constraint Language (OCL) specification, a formal language that is useful for defining constraints and conditions in software systems. This ensures a level of reliability and consistency in the composition of IoT services. Share allows the composition of services for IoT devices that have limited resources in terms of memory and computing power. IoT devices have limited resources, and they exchange integration codes that specify the data format and communication protocol. This allows the hardware limitation to be overcome. Furthermore, this overcomes the limitations of data-oriented standards and enables the dynamic composition of services. Through the use of a matching language, Share offers the possibility of finding services (for composition purposes). To ensure that matched services confirm the limits imposed by composition, the design-by-contract scheme (DCS) [14] is used. The Share pattern introduces two key innovations: it can operate on tiny devices with limited resources, and it allows the composition of peer-to-peer services without the need for a central organization. The explicitly defined LUA programming language was used to implement Share. ESP32-embedded devices were used to test this implementation. LUA was chosen as the programming language because there is a version optimized for microcontrollers with limited resources, 16 KB RAM, and 128 KB Flash [15].

### Related Work

It is well known that IoT devices have limited computational resources, so data-oriented integration approaches such as WSDL [10,11,12] are very complicated to apply. The reason for this is related to the requirement of [16] validators to verify the compatibility of services. Due to limited computing and memory resources, and due to the complexity of the validators, it is not possible to run them on embedded devices. Furthermore, it must be pointed out that validators based on first-order logic are required, so complex constraints on service integration cannot be verified in WSDL [17,18,19]. During a call to verify that service constraints are satisfied, one might think that a reasonable approach would be the design-by-contract (DCS) [14] scheme. By adding preconditions, postconditions, and invariants to the standard definition of abstract data types, DCS requires programmers and designers to create formal, accurate, and verifiable interface specifications for software components and call these specifications contracts. The use of theories of Satisfiability Modulo allows the verification of the compatibility of the caller’s specifications with those of the called. For this reason, there are different tools (e.g., Boogie [20] and Z3 [21]) that allow the verification of constraints. To avoid problems such as the explosion of states to which these approaches are subject, a posteriori constraint verification is adopted to have a more efficient review. It has been demonstrated that no prior verification is performed during the invocation of the service. In the event of a failure during the invocation phase, it is understood that the issue is related to the caller’s preconditions not meeting the requirements of the caller. Conversely, if failure occurs during the review of the caller’s preconditions, then what does not satisfy the caller’s preconditions is the result of the call. This type of approach is also used by Share.

In [22], the OSGi specification is described, which concerns a complete and dynamic component model via a modular system implementing a service platform for Java. In the OSGi specification, we find applications or components that are bundled and can be installed, started, upgraded, uninstalled, etc., remotely without the need to reboot. Currently, they are widely used in applications ranging from smartphones to cars, industrial, marine, and building automation, PDAs, entertainment, and application servers. It might seem that a similar approach is impossible to replicate on sensor devices, given their limitations in terms of memory and computing power. Small devices may not have the resources needed to run a JVM and any other OSGi services. To give an example, an ESP32 microcontroller costing a few dollars would not be able to run an OSGi framework. The Share pattern, on the other hand, can be executed directly on a device with limited resources that is itself a node. On an ESP32 microcontroller [23] via the LUA programming language, we successfully executed Share. Devices implementing Share can communicate in peer-to-peer mode without the need for additional components.

In their comprehensive study, Washizaki et al. [24] present a detailed survey of IoT system design and architecture models that offer a structured approach to addressing typical IoT constraints, enabling their analysis from a design-oriented perspective. Their classification of reusable solutions contributes to improving both interoperability and efficiency in resource-constrained environments. To support the standardization of interactions between hardware and software, requirement patterns play a key role in defining shared constraints and functionalities.

Similarly, design patterns [25,26] based on embedded systems can offer effective models for structuring interactions between hardware and software. Approaches such as Share align with these methodologies by enabling flexible integration that adapts to the constraints typical of IoT environments. This adaptability supports service interoperability by design. Unlike traditional approaches that rely on pre-deployment verification, Share employs a runtime validation mechanism, allowing it to dynamically respond to changing system conditions.

In recent years, some works have been conducted in an integrated simulator for parallel and distributed systems [27]. In the case of our emulator, it does not allow us to interact in real time with the individual components to perform queries. The emulator developed in LUA with components that instantiate the Share pattern in LUA simply allows a completely free analysis of the system in all its components.

Our Share model and its emulator contribute to ongoing research on adaptive architectures and reliable service orchestration for IoT systems. Xu et al. [28] propose a study on protocol adaptation and conduct evaluations for the development of a 5G-based industrial wireless controller prototype. This highlights how an emulator, such as Share, could be valuable for testing configurations and behaviors before implementing real solutions. This is true both in industrial settings and in low-power IoT networks in general. The same author [29] proposes an architecture to improve networking within the industrial internet. Our approach is in line with this vision due to the interoperability between devices and services, but their focus is on high-demand industrial environments. Share is aimed at lightweight devices with limited resources. Share offers a complementary perspective on flexible and dynamic IoT integration.

In Section 2, we introduce the Share design model to illustrate the close relationship between this work and the previous one. Section 3, “Materials and Methods,” provides a comprehensive structural overview of the emulator and its implementation. Section 4, “Results,” demonstrates the use of Share with the simulation and Object Constraint Language (OCL). Section 5, titled “Discussion,” methodically evaluates the advantages and disadvantages of using Share, before providing a thorough conclusion and future work (Section 6) to the article.

## 2. Background

To better understand how Share operates and how it is simulated, it is essential to define both the structural and behavioral aspects of the design pattern. These aspects are effectively represented through class and sequence diagrams, which illustrate the interactions among components and the mechanisms enabling the system’s dynamic service composition.

Class diagrams are used to represent the static structure of the system, including key classes, attributes, and relationships. Sequence diagrams, on the other hand, capture the dynamic behavior by modeling time-ordered interactions between components. Together, these metamodels form the foundation for implementation, supporting an organized and scalable architecture.

**Figure 1:**
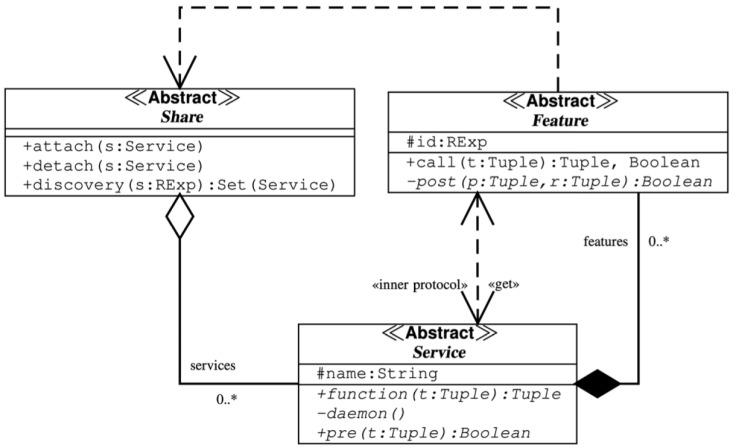
Share: class diagram.

In the figure above Figure 1, the class diagram of the Share design pattern is shown. As can be seen, it consists of the following classes:

- Share;
- Service;
- Feature.

*Service* object can be stored inside the space implemented by the class *Share*. This class has three public methods: *attach(s: Service)*, *detach(s: Service)*, *discovery(s: RExp):Set(Service)*.

The public method *attach(s: Service)* can be used to add a *Service s* to a *Share* object, while the method *detach(s: Service)*, on the contrary, allows removing the *Service s*. The third method *discovery(s: RExp):Set(Service)* can search all *Service* objects with a regular expression *s*. A set of *Service* that matches the regular expression *RExp* is returned to the caller.

For instance, a Service *double temperature_alert(double temp, double threshold)* can be defined to evaluate whether a given temperature value exceeds a predefined threshold and triggers an alert if necessary. This can be added by using the method *attach*. The service is identified by the string “2.5.8.10” to identify the specific function. It is possible to search a service, in this case, the service for the temperature alert, using a regular expression *RExep = “2.5.8.10”* when the *discovery* service is used. Figure 1 illustrates the *Service* class, which defines each service with a unique *name* acting as its identifier. This name can be treated similarly to a Management Information Base (MIB) entry in the Simple Network Management Protocol (SNMP). The MIB specifies an Object Identifier (OID) describing the function provided by the service [30]. In our example, we assign the MIB “2.5.8.10” to the *temperature_alert* service to uniquely identify it within the system. A *pre* predicate is defined by a *Service* class. This method takes as input a *Tuple t* and returns a boolean value. The tuple *t* can include the list of parameters that are taken as input by the service. The predicate should return true when the tuple *t* verifies the preconditions required to run the service *false* otherwise. In our *double temperature_alert(double temp, double threshold)*, the precondition predicate ensures that both temperature and threshold are within the valid operational range of the sensor. For instance, *boolean pre([temperature, threshold])* verifies that they are positive and within the sensor’s limits. IoT devices (such as ESP32-based sensors) can dynamically attach or discover this service using Share, enabling seamless interoperability among different sensor nodes. A *Service* class also needs to specify a *Tuple function(t Tuple)* and a *daemon()*. To interact with the *daemon*, the *function* is used as a connector, and it is sent to the client requesting the service. More specifically, *function(t Tuple)* acts as a client stub and converts the service parameters *t* into a format which the corresponding *daemon()* method can interpret. The *function* method establishes a connection, transmits the data to the *daemon*, and may optionally wait for a response. It is important to note that the serialization and deserialization handled by *function* and *daemon* are unnecessary when both the client and server use the same programming language and run on identical hardware. This setup enables highly efficient data transfer, particularly for handling complex structures or large data volumes. Additionally, when both components share the same language, it facilitates consistency checks and improves reliability, simplifying the use of verification tools. The class *Features* is the most important block in the design pattern of Share. To implement *Service* class behavior, the *daemon* can compose one or more *Features*, and its regular expression *id : RExp* describes the functionality that is implemented by the feature. This will be utilized to locate the required feature when a Share object is called. In practice, a service that has already been defined and added to a *Service* repository is defined by a *Feature*. The output of a *call* may be either *Boolean* or *Tuple*. The output of the *call* functionality is a *Tuple*, and, when the call execution ends successfully, the *Boolean* value is *true*; otherwise, it is *false*. This may result from a service not found error, a connection issue, or other kinds of failures specified inside the *call* implementation. A *post* predicate is defined by a *Features* class. This function returns a *Boolean* value after receiving a *Tuple t* as input. When the program evaluates the post-conditions after executing the *call*, the predicate should return *true*; otherwise, it should return *false*. In our example *double temperature_alert(double temp, double threshold)*, *Feature* could implement the validation check to make sure that the temperature does not pass over the given threshold before triggering an alert. In this case, the *call* takes the *Tuple [temperature, threshold]* and returns a *Boolean* value to indicate if the alert should be activated. This mechanism enables the system to integrate a new alerting service dynamically based on predefined conditions. In this way, it ensures efficient monitoring in IoT-based environments.

There are various Object Constraint Language (OCL) rules applied to the class diagram in Figure 1, and they are presented in Listing 1. Analyzing context rules, there are three methods previously discussed: *attach*, *detach*, *discovery*. The first context rule specifies the method to add the service *s* into the *services* association, while the second context rule specifies the method to remove the service *s* from the *services* association. The third context rule specifies the method that returns all services that match the regular expression *RExp s*. At the end, the last rule specifies the absence of duplicated services.

**Listing 1.** Share: OCL rules.
context Share::attach(s:Service)

  pre: services->excludes(s)

  post: services->includes(s)


context Share::detach(s:Service)

  pre: services->includes(s)

  post: services->excludes(s)


context Share::discovery(s:String):Set(Service)

  post: result = Set(services->select(name.matches(s)))


context Share

  inv: services->asSet()



To ensure consistency between the operations required to call an operation, OCL rules in Listing 2 must be followed. The results of the call are specified by the sequence *found* and defined by applying to all the services found with the discovery operation *pre*, *function*, and *post* using the tuple of the function *call*. The correct relation between parameters and operation (*pre*, *function*, and *post*) is defined by the rules definition. Finally, the result of the *call* operation is the first result of the *found* sequence or the couple: boolean, tuple, where the *true* value specifies the successful execution of the call invocation (i.e., a service has been found).

**Listing 2.** Share: feature OCL rules.
context Feature::call(t:Tuple):Boolean, Tuple

   def: found : Sequence(Service) =

     select(s : Share.discovery(id) |

       let s.pre(k:Tuple):Boolean, self.post(v,q):Boolean, s.

          function(w):r in

         t.isOclType() = k.isOclType() and

         t.isOclType() = v.isOclType() and

         r.isOclType() = q.isOclType() and

         s.pre(t) and self.post(t,s.function(t)))

    post: if found->notEmpty()

         then result =  {true, found->first().function(t)}

         else result = {false, Sequence{}} endif



In Listings 3 and 4, the implementation of a *Share temperature_alert* is represented with *LUA* language implementation. The *Service* in the example called *temperature_alert* takes the following parameters:

- the unique identifier of the *Service* (i.e., its MIB);
- a LUA function, i.e., the *Service function* implementation of the client stub;
- a LUA function that implements the *daemon*, which declares different parts:
  1. a *feature* with *RExp* 2.5.8.4.*;
  2. a *post* condition that specifies the comparison of the temperature and the threshold with precision;
  3. the reception of the parameters and the call of the *temp_check* function.
- the *pre* condition of the *temperature_alert* service, specifying that the temperature must be within the range of −50 to 150, inclusive.

On the other hand, in Listing 4, there is the *temperature_check Service* which takes as input the following parameters:

- the unique identifier of the *Service* (i.e., its MIB);
- a LUA function, i.e., the *Service function* implementation of the client stub, to enable a connection to the service;
- a LUA function which implements the *daemon*; here, it is possible to observe the validation of the comparison from the temperature and threshold;
- the *pre* condition of the *temperature_check* service, specifying that the temperature must be in the range −50 to 150 with edges included.

In Listings 3 and 4, it is possible to observe that they use the *attach()* method and register the newly created service to a *Share* object.

**Listing 3.** Share: service temperature_alert.
temperature_alert = Service.new("2.5.8.3.1", -- *declaration service*

  function(temp, threshold) -- *function*

  --*send temp and threshold to daemon and receive result value*

  end,

  function() -- *daemon*

    local temp_check = Feature.new(

      "2.5.8.4.*", --*RExp*

      function(temp, threshold)

        return temp >= threshold

    end --*postcondition*

     )

    --*receive parameters from function using inner protocol*

    local alert, ok = temp_check.call(temp, threshold)

    --*send result value alert to function using inner protocol*

  end,

  function(temp, threshold)

    return temp >= -50 and

    temp <= 150 and

    threshold > 0

  end

) --*precondition*

deviceA = Share.new(myIp)

deviceA.attach(temperature_alert)



**Listing 4.** Share: service temperature_check.
temperature_check = Service.new("2.5.8.4.1", -- *declaration service*

  function()

    return function(temp, threshold, ip)

      local tcp = socket.tcp() -- *Initialize tcp socket*

      local host, port = ip, 8888

      tcp:connect(host, port)

      tcp:send(temp .. ’,’ .. threshold)

      local result, status = tcp:receive() -- *Get response once*

      tcp:close() -- *Close connection*

      return result

    end

  end,


  function()

    local server = socket.bind("*", 8888)

    while true do

      local client = server:accept()

      local data, err = client:receive()

      if not err then

        local temp, threshold = data:match("([^,]+),([^,]+)")

        temp, threshold = tonumber(temp), tonumber(threshold)

        if temp and threshold and temp >= threshold then

          client:send("ALERT: Temperature exceeded threshold!")

        else

          client:send("Temperature is within normal range.")

        end

      end

      client:close() -- *Close client connection*

      -- *No break here to allow continuous operation*

    end

  end,


  function(temp, threshold)

    return temp >= -50 and temp <= 150 and threshold > 0

  end

)


deviceB = Share.new(Myip)

deviceB.attach(temperature_check)



### Sequence Diagram

It is imperative to acknowledge the pivotal function of sequence diagrams in modeling the interactions between disparate entities within a system. The utilization of these tools facilitates the representation of message exchange and the sequence of operations. This makes them important tools for understanding and verifying the behavior of complex service-based architectures. In the context of this article, in the sequence diagram, it is possible to understand how *Share* dynamically invokes services according to predefined patterns. This ensures a seamless interaction between the distributed components.

**Figure 2:**
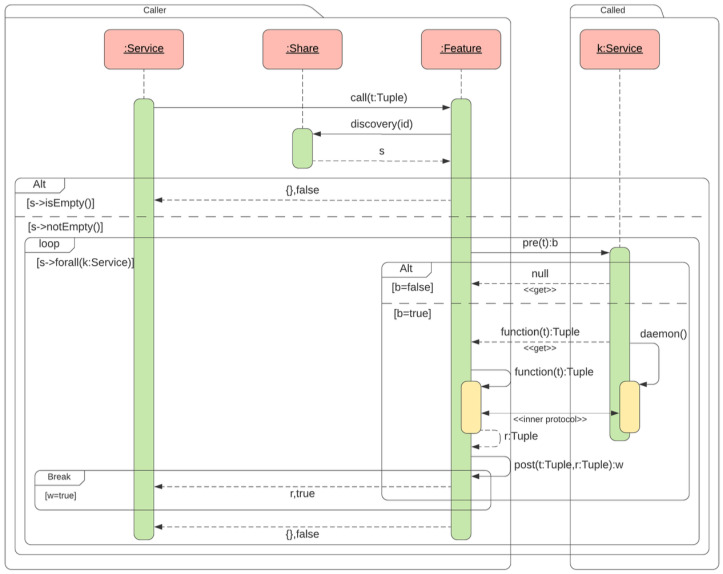
Share: service call sequence diagram.

In Figure 2, the sequence diagram highlights the interactions between objects during a call to a *Service*, in our case, the *temperature_alert* service example. The process begins when the service *temperature_alert* calls the method *call* of the object *Feature*. This object searches for a suitable service using an ID that corresponds to a certain pattern defined by *RExp* (Regular Expression). This is achieved through the message *discover(id)*, which returns a set of *S* objects *Service* matching the identifier.

When no matching service is found, *S* is empty and an error is returned. In contrast, when a service is found, the available one is evaluated sequentially in a loop, as is possible to observe inside the sequence diagram Figure 2. Each service found has to follow a sequence of steps. First, a *precondition* check is performed (*pre(t): b*) to verify that the given input parameters satisfy the service requirements. Each time the *precondition* of a service fails, the next one is checked until the *precondition* is satisfied. The *daemon* is invoked by the *feature* object only if the service implementation is valid, and it uses a function stub for the call. The interaction between caller and callee via *daemon* takes place using the same programming language. The language can be LUA, as in our case, or another lightweight and interpreted language that allows introspection. As a result, the programming language becomes the protocol. The called sends to the caller a function that handles communication between who is requesting the service and who is providing the service. The data is exchanged between the caller and the callee using an *inner protocol defined or used by the programmer, as shown in Figure 1 and Figure 2. The code sent is a function that can be executed and inspected by the called party through reflection.*

In our case, analyzing the *temperature_check* when the service is invoked, it evaluates the temperature and checks if it exceeds the threshold. Then, the service returns the result *r: Tuple* and a status *w: boolean*. The execution of the service is considered successful when the *w* returns true and the *Service* call concludes. On the contrary, as mentioned above, the system continues to search for alternative services to meet all the conditions. This structured approach ensures flexibility and robustness, and allows *Share* to dynamically adapt to different service implementations while maintaining a consistent execution flow.

## 3. Materials and Methods

The goal of this work is to develop an emulator capable of replicating the behavior of IoT devices that can implement the Share model. It is important to clarify the difference between an emulator and a simulator: generally, a simulator only models abstract behavior or performance under theoretical conditions; an emulator is capable of recreating the actual runtime environment. This allows us to test the real interactions between devices and services with the developed code. This approach is fundamental for validating the feasibility of Share on devices with limited resources. This means that the implementation can be run on a real hardware device with minimal modifications.

Emulators are widely used in embedded systems and IoT research to bridge the gap with theoretical models or hardware prototypes [31,32]. To verify the correctness of LUA coroutines, the execution of OCL constraints, and attach/detach operations, the emulator described in detail below is used. Correctness is a necessary check to see how Share can work in a real deployment. This gives greater confidence in the interoperability and reliability of the proposed project.

### 3.1. Emulator Implementation

A dedicated emulator was developed to validate the effectiveness of Share and test its applicability in resource-constrained IoT contexts. The aim is to verify how the design pattern manages the dynamic composition of services and ensures integration between heterogeneous devices. The emulator’s emphasis on small devices with limited resources is predicated on the understanding that these devices are the most vulnerable. These frameworks are not conducive to optimal performance when utilizing substantial hardware resources. Consequently, the Share design pattern has been developed to circumvent the utilization of substantial frameworks that impose a significant burden on resources. This pattern employs lightweight on-the-fly communication, predicated on a particular programming language. The LUA programming language is employed as a use case in the code example provided in the paper.

It is important to note that the programming language need not be LURA. Share can also be implemented in other languages, such as C/C++, Python, and so forth. This phenomenon can be attributed to the emulator’s adherence to a specific design pattern, which establishes a set of precise guidelines. At the implementation level, the use of specific language is not required; the sole requirement is adherence to the rules of the design pattern.

The choice of programming language should fall on lightweight languages, such as LUA in our case, to facilitate use on systems with limited resources (RAM, CPU, flash, etc.). Choosing a language that supports source code introspection allows the caller to analyze the entire implementation of the invoked function at runtime, and not just its signature.

The emulator, implemented in LUA, enables the execution and monitoring of interactions between devices, simulating realistic environments. In addition, it exploits the Object Constraint Language (OCL) specification to ensure that composition constraints are respected, thus increasing the reliability of the system. The development of the emulator is facilitated by the utilization of the LUA programming language [33].

The choice of this programming language is due to several characteristics that make it perfect for implementation on embedded systems with limited resources. It is an efficient programming language with a very low weight (about 220 KB). This characteristic makes it ideal for the systems with which we are dealing. It is a multi-paradigm language capable of handling procedural, functional, and object-oriented programming, and this characteristic allows it to adapt to any context and development requirement. It is a very portable language because it is able to run on UNIX, Windows, IOS, Android, and, above all, microcontrollers. The source code of the mentioned platforms is similar because LUA follows the ISO (ANSI) C standard, and, thanks to this feature, there is no need to adapt the code to new environments, only the need for an ISO C compiler. It would be important to emphasize that, when validating a simulation on a real system with the Share code written in LUA, it allows the reuse of the same code on embedded systems with certain precautions. For example, when reusing the simulation code in a real system, the communication-related parameters that differ from the simulation should be changed, but the code as a whole remains the same.

**Figure 3:**
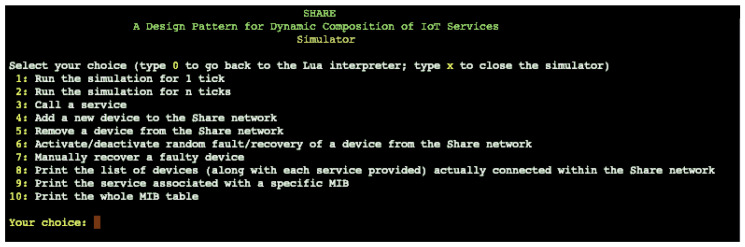
Share: emulator view from terminal.

The emulator is run from the terminal via the LUA compiler and is presented as seen in Figure 3. The emulator offers different possibilities for use, which are indicated and usable via a numbered list from 1 to 10. The first two commands (1, 2) allow one to simulate 1 to n ticks to see the effects of the simulation over some time of choice. Next in the list, we have the interactions with the *Share network*, where it is possible to call a service (4), add or remove a device from the network (5, 6), manage issues that may arise, and see how the system reacts through simulation (6, 7). Finally, with the last items in the list, we have the possibility of displaying the information of the Design Pattern Share by printing out the list of devices, the services with their MIB codes, or the entire table with the MIB codes.

### 3.2. Structure and Functionalities

As was the case in the preceding study [13], the present study places its focus on the example of the ideal climate inside a house. However, the present study analyzes the simulation instead of the generic case study defined by Baruch Givoni with his bioclimate-based graph [34]. The emulator, written in LUA, uses coroutines via the standard LUA Coroutine library to facilitate portability and simulate the parallel execution of callers and callees. Coroutines in LUA allow functions to interrupt and later resume the execution of the entire program without blocking it entirely, thanks to its lightweight and powerful mechanism for cooperative multitasking. While these coroutines may be regarded as analogous to conventional threads, they are distinct in their execution: coroutines share the same execution thread, and their operation is explicitly programmed by the programmer. This characteristic makes them particularly suitable for simulations and asynchronous control flows. The implementation of coroutines in LUA is regarded as a first-class value, signifying that they can be treated in a manner analogous to any other value, such as a number or string. Consequently, it is feasible to create a coroutine and store it within a variable or to pass it to a function. This feature allows developers to manage the execution state with minimal overhead, meaning that each coroutine keeps track of where it was paused (*coroutine.yield()*) to resume it at another time (*coroutine.resume()*). This feature makes it possible to pause and resume pieces of code with less complexity and resource cost than threads, making it perfect for implementation on systems with limited resources [35].

In our emulator, the *Service* has been implemented using LUA coroutines, as can be seen in the example Listing 5. The *Service* returns a temperature value with 2 decimal digit precision, and, in Listing 6, there is the implementation of the class definition.

**Listing 5.** Share: temperature sensor service.
-- *This Service returns a temperature value with 2 decimal digit precision*

coTemperatureP2 = coroutine.create(

    function()

        local t

        while true do

            t = intT + math.random(-100, 100)/100

            t = t - t%0.01      --*P2 precision*

            local tuple = {}

            table.insert(tuple, t)

            coroutine.yield(tuple)

        end

    end

)


temperatureP2 = Service:new(

    "temperatureP2",

    "1.1.2",

    [[ return function()

        status, values = coroutine.resume(coTemperatureP2)

        end

    ]] ,

    coTemperatureP2,

    function()

        return true       --*Pre-Conditions always satisfied*

    end

)



**Listing 6.** Share: definition of Service class.
-- *Definition of the Service~class*


Service = {}

Service.__index = Service


-- *This function implements the constructor of a Service object*

function Service:new(pMName, pName, pFunction, pDaemon, pPre)

return setmetatable({

    mName = pMName,      -- *Mnemonic name*

    name  = pName,      -- *MIB*

    sFunction = pFunction   -- *Service function*

    daemon = pDaemon       -- *Service daemon*

    pre = pPre         -- *Pre-conditions*

    }, Service)

end



Thanks to its modular structure, the emulator makes it easy to add new *Services* and their associated MIBs. Once the new *Service* has been added, it is possible to use it immediately in the emulator and go on to analyze the new scenarios that can be created with the use of the new service. The *Share network* is realized with a table where the keys are identified by their associated MIBs and the values are tables of *Services*. If we consider a real usage scenario, the mnemonic name can be the IP address or any other identifier that might work in a specific context. It is important to say that the values of *Share network* are tables of tables because each device within the network may offer more than one service and because LUA does not know the concept of class. A salient feature of the emulator is its capacity for a seamless transition between the simulation itself and the user agent. It is possible to pause the current simulation and resume it later without losing the current state. This can be achieved by switching to the interpreter and modifying the conditions in the environment in which *Share* is executing the simulation. Switching between the simulation and the user agent only performs a pause, so it is possible to start the simulation, make parameter adjustments, and then return to the simulation again and pick up where it left off.

The emulator focuses on those solutions that require common equipment such as humidifiers, dehumidifiers, air conditioners, boilers, pellet stoves, heat pumps, etc. These appliances belong to the category of equipment that can be switched on or off, and each actuator can provide from one to several *services*. In the context of the ideal indoor climate introduced by Baruch Givoni, it is necessary for equipment to function in certain ways to raise or lower the temperature until the ideal temperature is reached, also taking into account the outside temperature. Taking common appliances such as air conditioners and boilers into consideration and relating them to *services*, we can see how a boiler can offer only one *service* while the air conditioner offers two. The boiler allows the temperature to be raised, while the air conditioner also allows the temperature to be lowered and, optionally, the humidity to be lowered.

As mentioned earlier, when discussing the structure of the emulator and the services, we can group the *services* into categories via a semantic relationship where the actuators have their way of providing the *services* based on the European Union Energy Label [36,37]. For example, the service category decreasing humidity is identified as MIB 2.2.1.*, but within it there are several services related to the European label, as can be seen in Listing 7, which shows part of the MIB hierarchy table defined within the emulator.

**Listing 7.** Share: MIB table of emulator.
2.* : Actuators

  2.1.* : Temperature

    2.1.1.* : Increasing temperature

      2.1.1.1.* : Increasing temperature On

        2.1.1.1.1 : Increasing temperature On service (Energy Label A)

        2.1.1.1.2 : Increasing temperature On service (Energy Label B)

        2.1.1.1.3 : Increasing temperature On service (Energy Label C)

      2.1.1.2.* : Increasing temperature Off

        2.1.1.2.1 : Increasing temperature Off service (Energy Label A)

        2.1.1.2.2 : Increasing temperature Off service (Energy Label B)

        2.1.1.2.3 : Increasing temperature Off service (Energy Label C)

    2.1.2.* : Decreasing temperature

      2.1.2.1.* : Decreasing temperature On service

        2.1.2.1.1 : Decreasing temperature On service (Energy Label A)

        2.1.2.1.2 : Decreasing temperature On service (Energy Label B)

        2.1.2.1.3 : Decreasing temperature On service (Energy Label C)

      2.1.2.2.* : Decreasing temperature Off service

        2.1.2.2.1 : Decreasing temperature Off service (Energy Label A)

        2.1.2.2.2 : Decreasing temperature Off service (Energy Label B)

        2.1.2.2.3 : Decreasing temperature Off service (Energy Label C)

  2.2.* : Humidity

    2.2.1.* : Increasing humidity

      2.2.1.1.* : Increasing humidity On

        2.2.1.1.1 : Increasing humidity On service (Energy Label A)

      2.2.1.2.* : Increasing humidity Off

        2.2.1.2.1 : Increasing humidity Off service (Energy Label A)

    2.2.2.* : Decreasing humidity

      2.2.2.1.* : Decreasing humidity On

        2.2.2.1.1 : Decreasing humidity On service (Energy Label A)

        2.2.2.1.2 : Decreasing humidity On service (Energy Label B)

        2.2.2.1.3 : Decreasing humidity On service (Energy Label C)

      2.2.2.2.* : Decreasing humidity Off

        2.2.2.2.1 : Decreasing humidity Off service (Energy Label A)

        2.2.2.2.2 : Decreasing humidity Off service (Energy Label B)

        2.2.2.2.3 : Decreasing humidity Off service (Energy Label C)



The Share pattern by consulting the network, Share network, enables the *Service* to base the decision on how many devices are connected at the moment by calling the whole category and possibly changing the behavior according to the available *Services* within the category. In Listing 8, it is possible to see how the emulator has two different *Services* to achieve the ideal temperature according to Baruch Givoni’s comfort zone theory: generic and efficient. Both use the *services* provided by the sensors and actuators as their *features* and, in the first case, with the generic *service*, all available actuators within their categories are used with the sole purpose of reaching the desired comfort zone without thinking about energy consumption. Conversely, the efficient *service* is characterized by its consideration of energy efficiency and its utilization of actuators distinctly. This approach aims to maintain energy consumption at the level stipulated by the established label. The energy-efficient *service* tries to minimize the energy consumed; in particular, it tries to use services with an energy efficiency label A and only use them. If it is not possible to use only those with the energy efficiency label A, it uses actuators with the lower energy efficiency label (B or C).

**Listing 8.** Share: emulator Givoni.
4.* : Givoni

    4.1.* : Generic

        4.1.1 : Generic On service

        4.1.2 : Generic Off service

    4.2.* : Efficient

        4.2.1 : Efficient On service

        4.2.2 : Efficient Off service



For a complete simulation of a complex distributed network, there are also Listing 9 mathematical *services* that are used to perform calculations on the actual system, where again we can find a similar situation to that for actuators, of having a variable amount of services available. Depending on the devices providing the various services, we can have a system that provides only the average, only the minimum, or only the maximum. Other situations allow for only two services, while other situations allow for all services to be available. The behavior of the caller may be based on these factors, representing the *Share network* at a specific time.

**Listing 9.** Share: mathematics services.
3.* : Mathematics

  3.1.* : Average

    3.1.0 : Average service with an accuracy of zero decimal digit

    3.1.1 : Average service with an accuracy of one decimal digit

    3.1.2 : Average service with an accuracy of two decimal digits

  3.2 : Minimum service

  3.3 : Maximum service



Taking the service dealt with in Listing 3 as an example, its behavior can also be adapted to the scenarios that arise. If the *Service* needs to monitor the temperature to trigger a too-high (or too-low) temperature alert and only has one temperature sensor available, this service will have to rely on the only available *Service*. If, on the other hand, this service has more than one temperature sensor at its disposal, it has the option of using mathematical *Services* such as average, minimum/maximum, or all three. Consequently, the system’s elasticity can be discerned. In the particular instance of the emulator, the parameters can be modified by introducing (or eliminating) the *Services* to observe the system’s response. This adjustment does not compromise the progress that has been accumulated. In the case of mathematical calculations, it is possible to suspend the emulator to remove (or add) *Services* and see how the behavior of the system changes.

**Figure 4:**
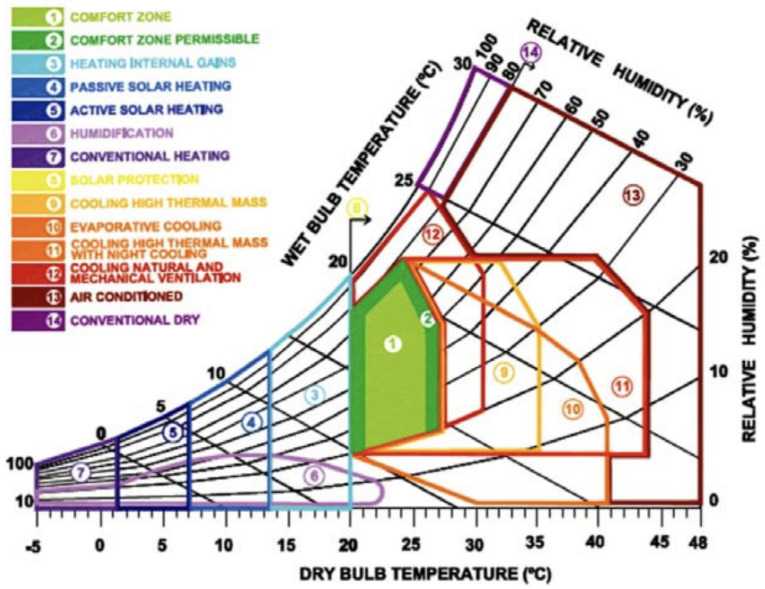
Baruch Givoni bioclimatic chart.

For the simulation, Figure 4, Baruch Givoni for bioclimate, was adjusted with the unit scale of degrees Celsius, replacing degrees Fahrenheit. It was then divided into zones, separating or joining one or more zones of the original chart. This was performed based on the consideration of which actuators are required to reach the comfort zone about energy consumption, as can be seen in Listing 10. For example, if one were to consider a temperature *T* of *T* = 16 °C and a humidity *H* of *H* = 20%, to reach a comfort zone, one would need to use an actuator that has a *service* that increases the temperature and another actuator that has a *service* that increases the humidity.

If one considers the case where *T* = 16 °C and *H* = 60%, the need for the system is only related to the temperature, and one uses an actuator with the *service* that increases the temperature.

**Listing 10.** Share: zones.
function aZone (t, h)

    return (t <= 26 and h <= 20 and h > -10/3*t + 260/3)

        or (t > 26 and t <= 41 and h <= -2/3*t + 112/3)

        or (h > -2/3*t + 112/3 and t > 26 and h <= -t + 76 and h <= -35/8*t + 185)

end


function bZone (t,h)

    return (t > 41 and h <= 10)

        or (h > 10 and h > -35/8*t + 185 and h <= -28/17*t + 1774/17

                   and h <= 80 and t > 33)

        or (t <= 33 and h > - 35/8*t + 185 and h <= - 10/3*t + 160)

        or (h > -t + 76 and h <= -35/8*t + 185 and h > -15*t +440 and h<= -5*t + 200)

end


function cZone (t,h)

    return (h > 22 and t > 20 and t <= 33 and h > -10/3*t + 160)

        or (h > 22 and t > 33 and h > -28/17*t + 1774/17)

        or (t > 25.8 and t < 26 and h> 71 and h < 72.1)

end


function dZone (t,h)

    return h > 80 and h > -5*t + 180 and h <= -5*t + 200 and t > 16 and t < 24

end


function eZone (t, h)

    return t <= 20 and h <= -5*t + 180 and h > -2*t +60



With Baruch Givoni’s bioclimatic graph, it can be seen that humidity and temperature are inversely proportional. During the simulation, we assume a closed environment, and this means that, applying mathematics, increasing the temperature leads to an indirect decrease in relative humidity. The absolute humidity, however, remains the same because we are talking about a closed environment. The estimate of the average proportion between the two is $\frac{\Delta H}{\Delta T}\approx -4.347826087$ and indicates that, as the temperature increases by 1 °C, you decrease the percentage of relative humidity decreases by 4 points. The considerations that are made in the closed room simulation are also made based on the variation in the temperature outside the building. In the absence of active actuators, the indoor temperatures exhibit a high degree of dependence on the outdoor temperatures. The greater the disparity between indoor and outdoor temperatures, the more pronounced the temporal variation, which is manifested in the emulator as a change in the unit of measurement, referred to as a *tick*.

The direct work on humidity is different because actuators, such as humidifiers and dehumidifiers, act directly on absolute humidity, while, on relative humidity, they act indirectly. In fact, by going to work on absolute humidity, relative humidity is also affected. Using an empirical estimate, the proportion between the change in relative humidity and temperature emerges as $\frac{\Delta H}{\Delta T}\approx -7$.

### 3.3. Formal Specification with OCL

In this subsection, we address the emulator specifications with the support of the OCL language, before concluding with results and discussions.

The *Object Constraint Language* (OCL) is a formal language that is used to describe expressions on UML models. In particular, it is used to specify constraints that cannot be represented directly using only graphical notations. OCL, which is introduced by the Object Management Group (OMG), is a part of the UML specification and assumes a very important role in model-driven engineering (MDE) to ensure accuracy and consistency within models [38]. The OCL allows developers to write expressions without side effects and allows the inclusion of invariants within classes, *-pre* and *-post* conditions on operations, and protections on transitions. Expressions are evaluated on model instances and do not change the state of the system. This makes OCL a declarative and secure language for the [39] specification.

To ensure the correctness and consistency of the service offered by the Share pattern, it is necessary to specify formal constraints with the use of OCL following the standard format ISO/IEC 19507:2012 [40] (OMG 2.3.1).

When a service is added to Share, the verification of the system is crucial to prevent its consistency from being compromised. We can point to two fundamental conditions for this purpose, which are represented by *liveness* and *safeness* Listing 11. The condition of *liveness* ensures that all the *Features* required by the *Service* are implementable. This means that, for each *Feature* within the *Service*, there must be at least one *Service* capable of providing it. The constraint related to *safeness* serves to avoid circular dependencies that may arise during the composition of the *Service*; this is to ensure that the chain at a given time ends.

**Listing 11.** Share OCL constraints: attach(s:Service).
context Share::attach(s:Service)


pre: services->excludes(s)


  pre liveness:s.features->forAll(f | discovery([f.id](http://f.id/)).notEmpty())


  pre safeness: s.fratures->closure(collect(discovery(id).fratures)->excludes(s)


post: services->includes(s)



When a *detach* operation is performed Listing 12, inconsistencies may be introduced into the system by breaking the conditions of *liveness* for other services. Before removing a *service*, it is essential to verify that the remaining *services* continue to satisfy their *liveness* constraints.

**Listing 12.** Share OCL constraints: detach(s:Service).
context Share::detach(s:Service)


  pre consistency: Share::allInstances().services.features->

                    forAll(f | discovery([f.id](http://f.id/))

                      .notEmpty())


  post: services->excludes(s)



Our Share pattern assumes that at least one instance of Share exists in the system, although multiple instances of it are possible. Each *Feature* object performs queries on the Share repository to find the *Service* that satisfies its functionality, and, in particular, there is no structural dependency between *Feature* and Share. This feature allows a certain flexibility of implementation in the creation of services, such as Publish/Subscribe, present in the MQTT protocol. To ensure ease of use, the implementation of Share could be bound to a specific architectural development. For example, in a smart home scenario, the Share pattern could be implemented as a singleton within a central device, such as a Wi-Fi router that supports SNMP services. Some home routers within the low-cost end of the market, such as the GL.iNet GL-AR300M16-Ext and GL.iNet GL-X750, have the OpenWrt operating system already pre-installed, and this Linux-based open-source operating system is optimized specifically for network devices [41]. Using OpenWrt, it is possible to execute customized user services directly on the router, making it possible to realize operations such as *attach* and *detach* as network-accessible services. This gives home automation devices the possibility of automatically registering with the Share service repository the moment that they connect to the network. The centralized management of Share on the router offers the possibility of monitoring the availability of devices by performing implicit *detach* operations in the event of a disconnect from Wi-Fi.

In the case of a home automation system, where all embedded systems are connected to the same Wi-Fi, the Share component resides in a router or Wi-Fi hub, as this is the only means of communication. This choice is not mandatory and, theoretically, each device can implement its instance of Share, resulting in an excellent fault-tolerant system. The implementation of Share allows nodes to query it with MIB strings that act as semantic descriptors of the service searched for via SNMP. Furthermore, if each node implements Share, it is possible to obtain a service similar to a cache service. Share could implement the proxy design pattern to search for a service through its Share.

## 4. Results

The Internet of Things (IoT) is made up of heterogeneous devices that have different characteristics and functionalities. These devices can be simple sensors or actuators that have very limited resources, which is why a major challenge in the IoT world is the integration and peer-to-peer communication of devices, avoiding the use of a centralized entity. The Share design pattern underscores the capacity to circumvent these constraints by virtue of its ability to operate on devices with constrained resources. This facilitates the dynamic composition of peer-to-peer services without the necessity of a centralized entity, thereby exemplifying a decentralized approach to network architecture.

To evaluate the proposed design pattern (Share), we developed an emulator using the interpreted programming language LUA. For the simulation, coroutines were implemented to manage interactions between services and IoT devices. The latter are emulated in such a way as to make them heterogeneous in order to compare them with a real situation. The emulator design follows the key features of the Share design pattern based on dynamic registration (*attach*) and secure removal (*detach*). In addition, another key feature is compliance with OCL properties.

We can demonstrate with the implementation of the Share pattern that it is a flexible and decentralized approach for the composition of services in a distributed system. By using formal constraints for services via the Object Constraint Language (OCL), which conforms to ISO/IEC 19507:2012 [40] (OMG 2.3.1), we can perform a check on the fundamental properties of the design pattern such as *liveness*, *safeness*, and *consistency* during the operations of *attach* and *detach* when the simulation is in place. As the number of devices added to the Share network increased, the emulator maintained stable performance and low overhead. Synthetic scenarios with different services demonstrated that the model based on the Share design pattern efficiently manages execution on configurations with limited resources.

Even when analyzing dynamic conditions, the simulation manages to confirm that the system can maintain operational integrity, handling situations such as device disconnections or service removals.

In addition, the possible implementation of low-cost OpenWrt router devices enables deployment and use in embedded environments and smart infrastructures. To illustrate its feasibility, we outlined how the same logic of the Share design pattern can be implemented using OpenWrt. This is an open-source Linux distribution for network devices such as routers. The implementation of this scenario has not been implemented in reality with Share, but we have provided an example that demonstrates how the logic of the design pattern can be executed with these routers. The example highlights how lightweight routers can handle the Share service logic in real IoT contexts.

Finally, the results confirm that Share can satisfy the conditions of formal correctness during dynamic service management. The potential of the design pattern is highlighted as being suitable for implementation on devices with limited resources and low energy consumption, in dynamic conditions with low computational cost.

## 5. Discussion

From the above results, we can confirm that the Share model is able to provide a robust and extensible infrastructure to manage distributed services in embedded systems. The use of regular expressions to perform the discovery, together with the OCL for applying behavioral properties, allows for a structured system that can adapt to dynamic situations. One of the most important aspects of Share is the way in which it provides the availability of services through the operations of *attach* and *detach*, which guarantee the continuity of use without letting control be centralized. Another element is the cooperative execution of the system via LUA coroutines that allows multitasking and minimal overhead, and this makes the simulation environment both technically efficient but also conceptually accessible. The potential for significant progress in this area may be found in the use of low-cost routers integrating the OpenWrt operating system. This integration facilitates the incorporation of the Share repository and enables direct communication through the Wi-Fi network by managing the devices’ entry and exit from the network.

In the Share emulator, we use a simplified protocol stack to adapt it to IoT systems with limited resources in our case study. The assumption on which we have also provided the example in Listing 4 is based on connection via a standard TCP/IP network. It is reinforced by the example of OpenWrt-based routers. When a device connects (*attach)* , it performs the connection operation by announcing its service metadata (unique identifier, function, preconditions, postconditions)—Listing 3. Other devices within the network can detect available services by querying Share via the operation *discovery.*

## 6. Conclusions and Future Work

In conclusion, in this article, we have presented how the Share design pattern can be emulated with its implementation in LUA. It represents an extension of our previous work on the Share model for the dynamic composition of IoT services. We have introduced an emulator capable of testing and validating the effectiveness of the pattern, allowing the implementation to be refined before actual production. The use of the Object Constraint Language (OCL) specification ensures consistency within IoT service compositions. With Share, we have seen how it is possible to emulate the execution and monitoring of interactions between heterogeneous devices, enabling their interoperability. This also highlights that it is not necessary to rely on rigid, centralized infrastructures. Our results confirm that this approach supports flexible integration scenarios in IoT contexts.

Future developments may be related to the emulator, which could be used for the following:

- verify the workload of the individual nodes and the entire system;
- determine the functioning of the system in the presence of failures or the unavailability of devices;
- help in the design of fault-tolerance systems with safeguard causes.

In particular, one could investigated the redundant use of devices that implement the Share class, for example, different access points, to eliminate the critical point of the single device that guarantees communication. This requires the implementation of more functionality and not just the analysis of the log file present in the emulator.

A subsequent research opportunity could involve the examination of the transition from the emulator to a real system. It is imperative to ascertain the viability of the present implementation, which is predicated on an emulated Share model, in a tangible system. The implementation of Share logic on actual hardware would facilitate the identification of practical values that may not emerge in a virtualized context. For instance, factors such as network latency, device power limitations, memory management, and unanticipated challenges may not be adequately addressed by an emulator. Consequently, a viable starting point for subsequent endeavors could be the implementation of authentic IoT nodes, such as ESP32-based sensors and OpenWrt routers. The validation and robustness of the Share design can be assessed through the reproduction of attachment/detachment operations and dynamic service discovery in a real environment. This approach would also facilitate the refinement of OCL constraints and code implementations, thereby addressing specific hardware limitations.

## Data Availability

Data is contained within the article.
